# Change in health spending after implementation of a health transformation plan in Iran: an interrupted time series analysis

**DOI:** 10.1186/s12962-021-00286-4

**Published:** 2021-06-03

**Authors:** Reza Esmaeili, Samad Rouhani, Jamshid Yazdani Charati, Masoud Khandehroo

**Affiliations:** 1 Department of Public Health, School of Health, Social Development and Health Promotion Research Center, Gonabad University of Medical Sciences, Gonabad, Iran; 2 Department of Public Health, School of Health, Mazandaran University of Medical Sciences, Sari, Iran; 3 Department of Biostatistics, School of Health, Mazandaran University of Medical Sciences, Sari, Iran; 4 Social Development and Health Promotion Research Center, Gonabad University of Medical Sciences, Gonabad, Iran

**Keywords:** Health expenditures, Health insurance, Health reform, Hospitalization rate

## Abstract

**Background:**

Health transformation plan (HTP) implemented in Iran since 2014 to improve accessibility and financial protection of patients. This study aimed to assess the impact of HTP on health spending in Iran.

**Methods:**

This was a quasi-experimental design using Interrupted Time Series. All registered impatient records in Iran health insurance organization (IHIO) for the population of Mazandaran province (1,628,919 population in 2011), north of Iran from March 2010 to February 2019 were included. Data for three depended variables: hospitalization rate, average inpatient cost and inpatient expenditure per capita was extracted in 96 monthly observations. Segmented regression analysis was done in R version 3.6.1.

**Results:**

Hospitalization rate in 2010 was 6.6 in 1000 people and its level change was 0/799 immediately after HTP (P < 001). Post-reform level and trend changes for monthly average inpatient cost of registered admissions in IHIO were also significant (P < 001). IHIO inpatient expenditure per capita for 1,628,919 population in Mazandaran province was 24,436 Rials in 2011 and increased significantly immediately following HTP as 34,459 Rials (P < 001).

**Conclusions:**

Three important components of health spending including hospitalization rate, average inpatient cost and inpatient expenditure per capita were increased dramatically after HTP. Cost containment strategies and strengthening the preventive care initiatives is required to control the escalating trends of inpatient expenditure in Iran.

**Supplementary Information:**

The online version contains supplementary material available at 10.1186/s12962-021-00286-4.

## Background

Moving toward Universal Health Coverage (UHC), in the context of economic crisis and limited resources, requires sustainable financing and more effective resource allocation mechanisms throughout the implementation of new health reforms [1]. Sustainable Progress in UHC needs to address the attributes of high-performing health systems: quality, efficiency, equity, accountability, sustainability and resilience in health reforms monitoring and evaluation [2]. Inpatient and day curative care accounted as largest share (35%) of public health spending at a global trend [3]. Specifically, over one-third of total English NHS expenditure [4] and 53.5% of the total medical cost in Medical-aid scheme of South Korea [5] devoted to inpatient care. So, changes in inpatient care volume, cost and expenditure will cause broad effect on overall health spending.

Reforming public hospitals for improving access to health care and decreasing health inequality were done in some countries such as China [6, 7] and Iran [8] in the last decade. Post-reform health spending monitoring are needed to examine the implementation and effects of these reforms.

**Fig. 1:**
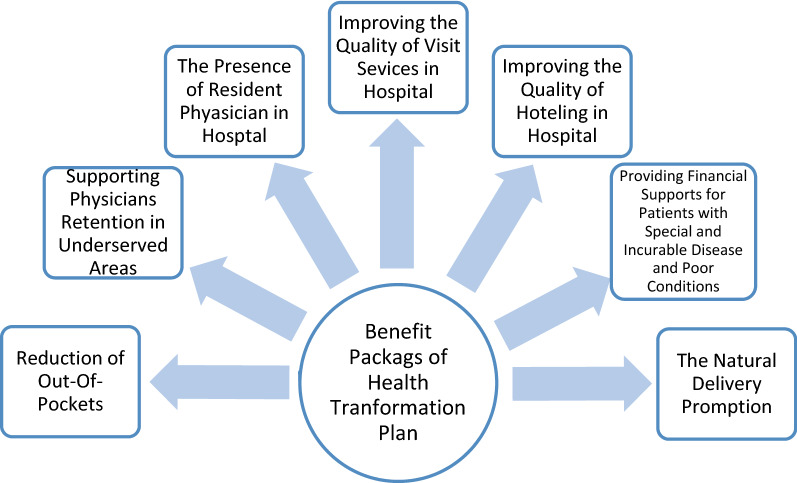
Framework of service packages of Health Transformation Plan (HTP) in Iran

Iran ministry of health has introduced HTP in 2014 with seven service packages (Fig. 1). HTP mainly focused to increase accessibility of healthcare and decrease out-of-pocket payments for secondary care in Iran health system [8, 9]. Through the three implementing phases, the medical tariffs increased dramatically [10] and out-of-pocket payments reduced noticeably in hospital settings [11]. So, the expected impacts of HTP on health care utilization and expenditure made uncertainties about health spending change and financing sustainability of Iranian health care system [12, 13].

For better understanding about changes in health spending after HTP in Iran, this study investigated empirical evidence about the level and trend of inpatient spending throughout three areas: hospitalization rate, average cost per inpatient and per capita expenditure using ITS analysis.

## Methodology

### Study design

This was a quasi-experimental design using Interrupted Time Series (ITS). ITS is the strongest quasi-experimental design for real world data of health policies and reforms evaluation [14, 15]. The outcome measures for the time series analysis were hospitalization rate (HR), average inpatient cost (AIC) and inpatient expenditure per capita (IEPC) of IHIO covered population across monthly intervals.

### Data sources

All registered impatient records in IHIO from March 2010 to February 2019 for the population of Mazandaran province, north of Iran were included in the study (Table 1). Data on three depended variables: HR, AIC and IEPC was extracted in 96 monthly observations.

**Table 1:** Segmented regression of time-series data for hospitalization rate per 1000 population

Parameter	Coefficients	Standard errors	t-statistic	Confidence interval		P value
Initial level	6.587	0.185	35.480	6.217	6.957	0.000
Pre intervention slope	− 0.015	0.008	− 1.831	− 0.016	0.016	0.070
Change in the level immediately after HTP	0.799	0.240	3.331	0.319	1.279	0.000
Change in slope after HTP	0.058	0.010	5.618	0.038	0.078	0.000

*HTP* Health Transformation Plan

### Data analysis

A linear regression model was applied as following equation.

$${\text{Yt}} = \beta 0 + \beta 1{\text{ time}} + \beta 2{\text{ interruption }}\left( {{\text{HTP}}} \right) + \beta 3{\text{ time after interruption }}\left( {{\text{HTP}}} \right) + {\text{e}}$$

In this model:

- β0 is the baseline level of the three outcome measures at the beginning of the time series.
- β1 is the slope before HTP.
- β2 is the change in level immediately after the HTP.
- β3 is the change in slope after HTP.
- e represents the value of residuals.

We used segmented analysis for estimating both immediate (level) and long-term (trend) impacts of HTP.

Several diagnostic assessment tests were done. First for detecting autocorrelation between residuals, Durbin Watson test was conducted which we corrected using the Praise Winston method. Augmented-Dickey-Fuller statistic was also used to determine the stationary of time series. To check the normality of the residuals we used Kol¬mogorov–Smirnov statistic. Bartlett test was used to assess the homogeneity of variance between residuals (Additional file 1). All analyses were done in R version 3.6.1.

## Results

### Change in hospitalization rate

**Fig. 2:**
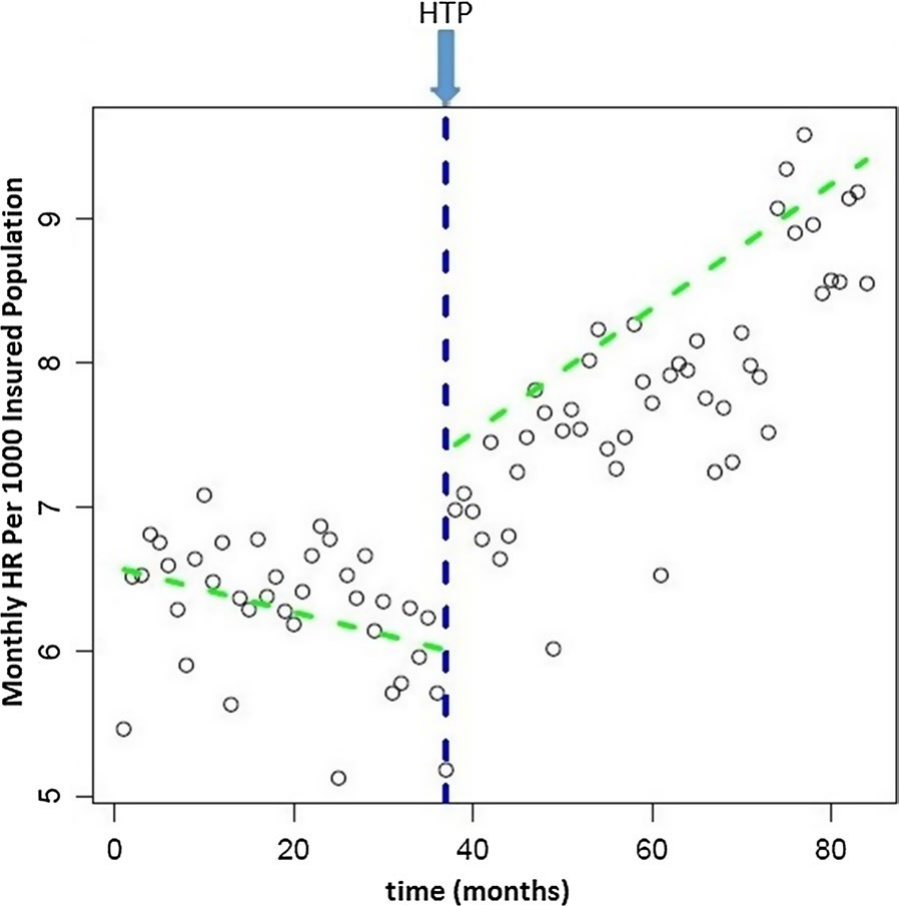
Level and trend of hospitalization rate per 1000 population before and after HTP

HR at the beginning of the study period (March 2011) was 6.6 in 1000 population and was downward. But a significant level change occurred immediately after the introduction of HTP (P < 0.001). Trend of hospitalization rate after HTP increased about 0/06 every month significantly (P < 0.001) (see Fig. 2 and Table 1).

### Change in average inpatient cost

**Fig. 3:**
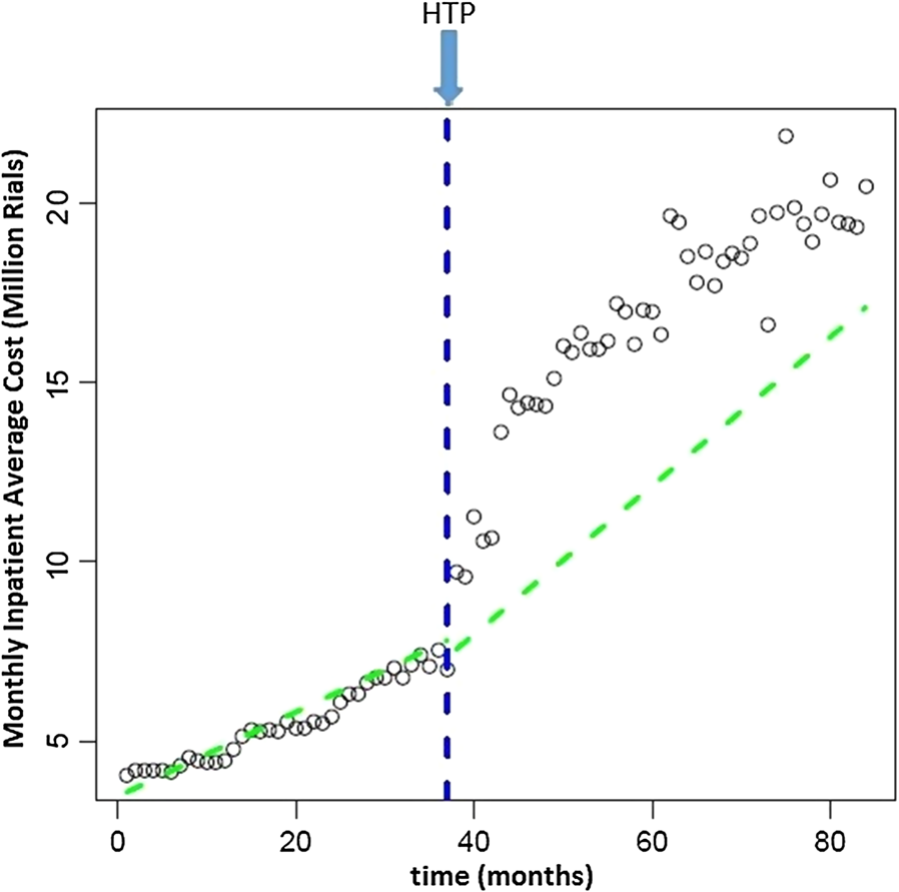
Level and trend of average inpatients cost before and after HTP

Time series of 96 monthly average inpatients cost from March 2011 to February 2019 are showed in Fig. 3. Initial level was 3,469,434 Rials and every month 117,549 was being added to the AIC throughout the years until HTP (Table 2). Change in the level of AIC immediately after introduction of HTP was 3,886,634. Change in monthly trend of AIC was also significant for the post-reform years (Table 2).

**Table 2:** Segmented regression of time-series data for average inpatients cost

Parameter	Coefficients	Standard errors	t-statistic	Confidence interval		P value
Initial level	3,469,434	615,894	5.633	2,237,646	4,701,222	0.000
Pre intervention slope	117,549	26,919	4.367	63,711	171,387	0.000
Change in the level immediately after HTP	3,886,634	676,910	5.742	2,532,814	5,240,454	0.000
Change in slope after HTP	89,530	35,812	2.500	17,906	161,154	0.014

### Change in inpatient expenditure per capita

**Fig. 4:**
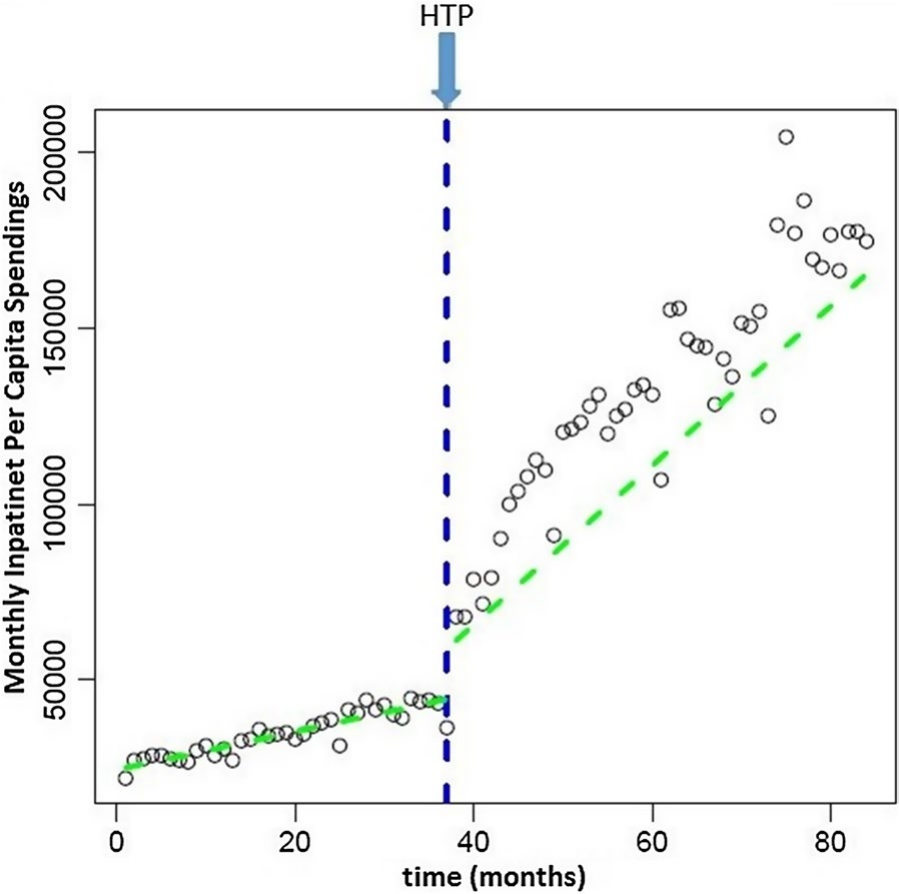
Level and trend of inpatient expenditure per capita

IEPC for 1,628,919 population in Mazandaran province was 24,436 Rials in 2011 from the perspective of IHIO. This level increased significantly immediately after the implementation of HTP as 34,459 Rials. Post-reform trend of IEPC increased significantly 1720 Rials each months (three times versus the trend before of HTP) (See Fig. 4 and Table 3).

**Table 3:** Segmented regression of time-series data for inpatient expenditure per capita

Parameter	Coefficients	Standard errors	t-statistic	Confidence interval		P value
Initial level	24,435.9	4521.7	5.404	15,392.5	33,479.3	0.000
Pre intervention slope	543.5	205.6	2.643	132.3	954.7	0.000
Change in the level immediately after HTP	34,459	5737.8	6.006	22,983.5	45,934.7	0.000
Change in slope after HTP	1720.9	255.9	6.726	1209.1	2232.7	0.000

## Discussion

Our findings showed HTP as the latest health reform in Iran, resulted in a significant increase in HR, AIC and IEPC from the health insurance perspective.

HTP targeted to less out of pocket payment in specialist outpatient clinics and inpatients settings. So, Increase in the HR can be interpreted as improving access to inpatient care in Iran. But the poor referral system, the prominent role of fee-for-service and lack of clinical guidelines weaken these interpretation. Potential over provision and over utilization during first years after HTP has been raised in similar literatures [12, 16, 18]. From a comparative perspective with china health reform, Tao et al. [19] mentioned achievements and challenges of 10 years of healthcare reform in China. They reported considerable increase in hospital admission and annual resident HR following reform. They noticed some challenges that surround China health system reform achievements. These challenges include fragmented healthcare delivery, hospital-centered and treatment-dominated. In such circumstance, both countries need to focus on reforms toward integrated and people-centered health system.

In addition to the increase of HR, AIC was increased compared to the prior years of HTP. In a similar study, Khadivi et al. [20]. Compared the direct health expenditures before and after the implementation of HTP in Isfahan Province. They reported a 1.49 to 2.3 times increase in direct health expenditure after HTP.

Finally, our findings indicated a significant upward level and trend of IEPC from IHIO perspective after HTP. As regards, social insurance is the greatest financing agent in Iran health system, these changes will create critical effect on overall health spending.

The limitation of this study is the lack of control group. Also Mazandaran province is a pilot site for establishing urban family physician plan in Iran, future additional studies in more diverse population is needed.

## Conclusion

Health spending in the term of AIC and IEPC increased dramatically after the introduction of HTP in Iran. This substantial increase of expenditure in the extensive hospital settings in Iran will push policies to high premium, high out of pocket payments and absorbing greater public spending. Regressive financing mechanisms for compensation of increased expenditure will thread health equity. Efforts to reduce inpatient expenditure in Iran, would follow investment on preventive care initiatives such as family physician, designing effective cost containment strategies and establishment of output-based payment systems such as Diagnosis Related Group (DRG). For future research, investigation on changes in health spending based on National Health Accounts (NHA) and households’ income-expenditure surveys will create clearer understanding of spending patterns and other potential explanations for the rise in health care expenditures. The relationship between health expenditure and health outcomes is also proposed to measure the overall effectiveness of these steady increases in health spending.

## Supplementary Information

**Additional file 1.** Statistical diagnostic tests.

## Data Availability

The datasets used in the study are available from the corresponding author on reasonable request.

## Abbreviations

HTP: Health Transformation Plan
IHIO: Iran Health Insurance Organization
ITS: Interrupted Time Series
UHC: Universal Health Coverage
HR: Hospitalization Rate
AIC: Average Inpatient Cost
IEPC: Inpatient Expenditure Per Capita
