# Supplementary material for: Change in health spending after implementation of a health transformation plan in Iran: an interrupted time series analysis
Source: Cost Eff Resour Alloc. 2021 Jun 3;19:32. doi: 10.1186/s12962-021-00286-4 (PMC8173793; doi:10.1186/s12962-021-00286-4)
Supplement: Supplementary file 1 — Additional file 1. Statistical diagnostic tests. [file 12962_2021_286_MOESM1_ESM.docx]

CERA-D-21-00021
Change in Health Spending After a Health Transformation Plan in Iran: An Interrupted Time Series Analysis
Reza Esmaeili; Samad Rouhani; Jamshid Yazdani-Charati; Masoud Khandehroo
Cost Effectiveness and Resource Allocation

Additional file:

Normality tests:

|  | HRP | AIC | IEPC |
| --- | --- | --- | --- |
| qq plot | 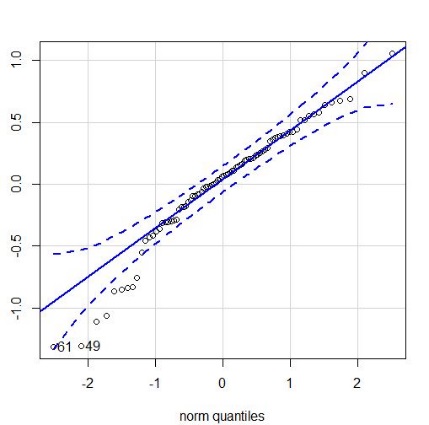 | 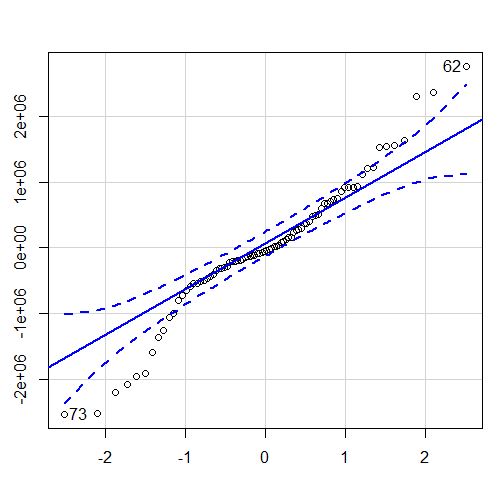 | 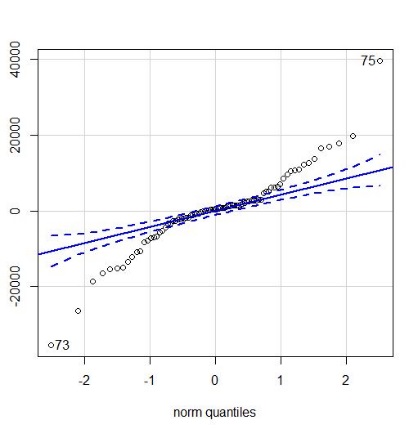 |
| Histograms | 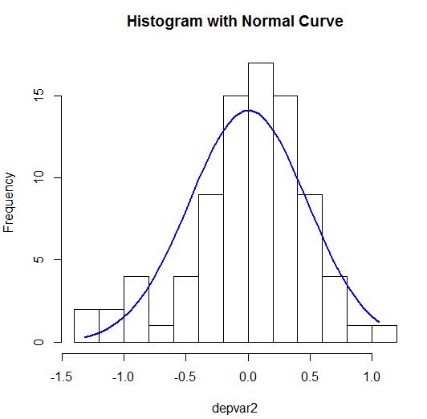 | 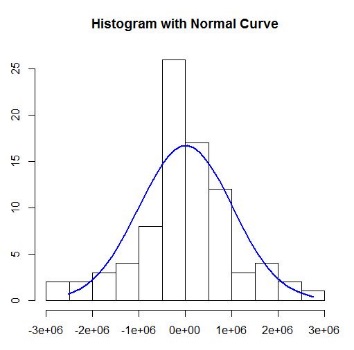 | 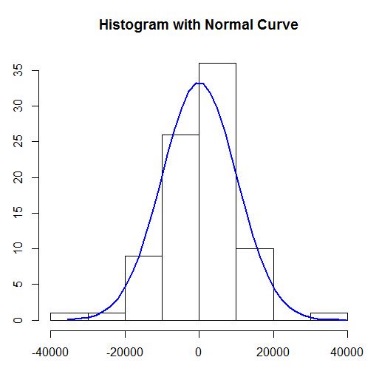 |
| Residuals over time (OLS) | 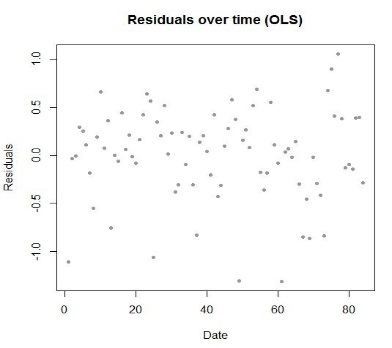 | 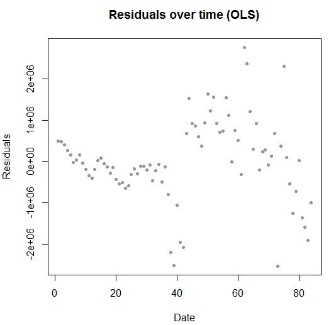 | 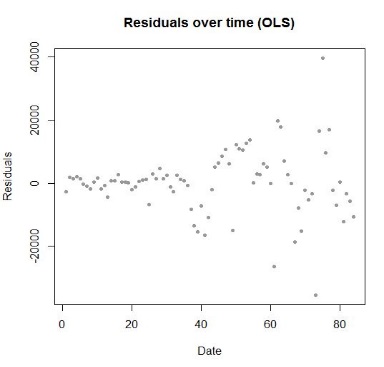 |
| One-sample Kolmogorov-Smirnov test | P<001 | P<001 | P<001 |
| Durbin-Watson statistic | original: 1.643  transformed: 1.903 | original: 0.8844  transformed: 1.972 | original: 1.407  transformed: 1.969 |
| F-statistic | 68.63 on 3 and 80 DF, p-value: < 0.001 | 239.4 on 3 and 80 DF, p-value: < 0.001 | 429.9 on 3 and 80 DF, p-value: < 0.001 |
| ADF | Dickey-Fuller = -3.8624, Lag order = 4, p-value <0.001 | Dickey-Fuller = -2.0669, Lag order = 4, p-value = 0.548 | Dickey-Fuller = -3.8661, Lag order = 4, p-value < 0.001 |

Engel and Granger co-integration approach

| Null Hypothesis: HRP has a unit root | | | |  |
| --- | --- | --- | --- | --- |
| Exogenous: Constant | | |  |  |
| Lag Length: 0 (Automatic - based on SIC, maxlag=11) | | | | |
|  |  |  |  |  |
|  |  |  |  |  |
|  |  |  | t-Statistic | Prob.* |
|  |  |  |  |  |
|  |  |  |  |  |
| Augmented Dickey-Fuller test statistic | | | -8.036805 | 0.0000 |
| Test critical values: | 1% level |  | -3.511262 |  |
|  | 5% level |  | -2.896779 |  |
|  | 10% level |  | -2.585626 |  |
|  |  |  |  |  |
|  |  |  |  |  |
| *MacKinnon (1996) one-sided p-values. | | | |  |

| Null Hypothesis: AIC has a unit root | | | |  |
| --- | --- | --- | --- | --- |
| Exogenous: Constant | | |  |  |
| Lag Length: 0 (Automatic - based on SIC, maxlag=11) | | | | |
|  |  |  |  |  |
|  |  |  |  |  |
|  |  |  | t-Statistic | Prob.* |
|  |  |  |  |  |
|  |  |  |  |  |
| Augmented Dickey-Fuller test statistic | | | -4.481306 | 0.0005 |
| Test critical values: | 1% level |  | -3.511262 |  |
|  | 5% level |  | -2.896779 |  |
|  | 10% level |  | -2.585626 |  |
|  |  |  |  |  |
|  |  |  |  |  |
| *MacKinnon (1996) one-sided p-values.   \| Null Hypothesis: IEPC has a unit root \| \| \| \|  \| \| --- \| --- \| --- \| --- \| --- \| \| Exogenous: Constant \| \| \|  \|  \| \| Lag Length: 0 (Automatic - based on SIC, maxlag=11) \| \| \| \| \| \|  \|  \|  \|  \|  \| \|  \|  \|  \|  \|  \| \|  \|  \|  \| t-Statistic \| Prob.* \| \|  \|  \|  \|  \|  \| \|  \|  \|  \|  \|  \| \| Augmented Dickey-Fuller test statistic \| \| \| -6.604178 \| 0.0000 \| \| Test critical values: \| 1% level \|  \| -3.511262 \|  \| \|  \| 5% level \|  \| -2.896779 \|  \| \|  \| 10% level \|  \| -2.585626 \|  \| \|  \|  \|  \|  \|  \| \|  \|  \|  \|  \|  \| \| *MacKinnon (1996) one-sided p-values. \| \| \| \|  \| \|  \|  \|  \|  \|  \| | | | |  |
